# Predictor role of COVID-19 anxiety on maternal competency with mediating role of mother-infant attachment: A study of structural equation modeling

**DOI:** 10.1016/j.heliyon.2022.e09973

**Published:** 2022-07-16

**Authors:** Zahra Mirzaki, Zahra Behboodi Moghdam, Mitra Rahimzadeh, Fahimeh Ranjbar, Sara Esmaelzadeh-Saeieh

**Affiliations:** a Alborz University of Medical Sciences, Karaj, Iran; b School of Nursing & Midwifery, Tehran University of Medical Sciences, Tehran, Iran; c Social Determinants of Health Research Center, School of Public Health, Alborz University of Medical Sciences, Karaj, Iran; d Nursing Care Research Center, Iran University of Medical Sciences, Tehran, Iran; e Social Determinants of Health Research Center, Alborz University of Medical Sciences, Karaj, Iran

**Keywords:** Competence, Maternal-infant attachment, Anxiety, COVID-19

## Abstract

**Introduction:**

COVID-19 pandemic is one of the factors that can increase anxiety and stress levels in pregnant women. Anxiety reduces the maternal-infant attachment. Mother-infant attachment can play an important role in maternal competency. The aim of this study was to predict effect of COVID-19 anxiety during pregnancy and the postpartum period on the maternal competency with mediating role of maternal-infant attachment.

**Method:**

A prospective longitudinal study was conducted on 253 pregnant women in the third trimester of pregnancy that referred to the health centers of Iran University of Medical Sciences and met the study inclusion criteria. Demographic questionnaire and the COVID-19 anxiety scale completed during the third trimester of pregnancy and after the delivery and Müller's mother-infant attachment and maternal competency questionnaire completed at 48 h and 6 weeks after delivery by self-report method. Smart partial lease square version 3 was used to assess the validity and reliability of the model and the relationship between the variables.

**Results:**

The findings of this study demonstrated that the COVID-19 anxiety during postpartum had a significant negative effect on the maternal-infant attachment (β = −0.183). Also, the maternal-infant attachment had a significant positive effect on the maternal competency (β = 0.48). Moreover, the conceptual model had good validity, reliability, quality and fit. And also the two variables of anxiety and mother-infant attachment together explained 25% of maternal competency.

**Discussion:**

Mothers experience higher levels of the COVID-19 anxiety during pregnancy and postpartum; therefore, it is recommended that particular attention should be given to the psychological support of pregnant women during the COVID-19 pandemic and quarantine. Also, the COVID-19 anxiety during the postpartum period had a negative effect on the maternal-infant attachment and competency, which necessitates the need for the support of mother-infant relationship and providing the online training to promote the maternal-infant attachment patterns and maternal competency during the COVID-19 pandemic.

## 1 Introduction

In January 2020, the World Health Organization (WHO) declared that there is a high risk of spreading the coronavirus disease 2019 (COVID-19) to other countries, and in March 2020, WHO declared the pandemic of COVID-19 [1]. The respiratory diseases due to serious physical problems and reduced quality of life in patients can lead to anxiety [2]. Pregnant women are more susceptible to respiratory diseases due to physiological changes in the respiratory system during pregnancy, which are associated with increased infectious complications and high mortality in them [3].

The COVID-19 virus can also lead to fetal complications such as intrauterine growth restriction, preterm delivery, spontaneous abortion, and even fetal death. Recent studies show that pregnant women appear to be more susceptible to severe COVID-19 illness compared to non-pregnant women [3, 4]. COVID-19 pandemic is one of the factors that can increase anxiety and stress levels in pregnant women. The psychological problems during the prenatal period are the most important health issues and high levels of stress during pregnancy are associated with the negative consequences for psychological and physical health of infants and parents [5]. Pregnant women who need more emotional support may lose this support due to the restrictions imposed the COVID-19 and reduced contact with their relatives. In some cases, some pregnant mothers may not see a specialist to control their condition and that of the fetus for fear of getting infected by the COVID-19 [6]. Many studies also emphasize that persistent anxiety during pregnancy can cause the irreversible effects on the fetus and the mother [7, 8].

Anxiety causes the mother's inappropriate responses to the fetus during pregnancy and reduces the maternal-infant attachment. Mothers who were less attached to their fetus have higher levels of anxiety and depression, which can result in pregnancy complications [9]. Psychological problems in mothers such as depression, anxiety and stress can severely damage mother child relationships. Postpartum care is very important for the development of the maternal-infant attachment [10].

The first hour after birth is a sensitive period for the development of the maternal-infant attachment. There is evidence that postpartum anxiety is associated with poor maternal compromise and low self-esteem and affects the maternal attachment [11]. Severe anxiety during pregnancy affects the maternal-fetal attachment and reduces the mother's ability to play the maternal competency [12]. Also, the maternal-fetal attachment can play an important role in accepting the maternal identity, future mother-infant relationship, and a child's growth and development [13].

Stress during pregnancy reduces the maternal attachment and is associated with impaired parenting behaviors [14]. Maternal attachment has a fundamental effect on the development of the baby and the maternal competency. Mothers –Infant's relationship affecting maternal behaviors and improve positive emotionality in infants [15].

COVID-19 has led to significant changes in almost every aspect of daily life. These changes may expose pregnant women, maternal –infant attachment and mother hood. And also there is not enough study about COVID-19 anxiety and maternal –infant attachment and maternal competency, aim of this study was to explore the effects of COVID-19 anxiety during pregnancy and the postpartum period on the maternal competency with mediating role of maternal-infant attachment using the structural equation modeling (SEM).

**Figure 1:**
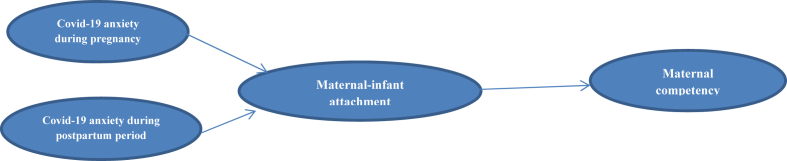
Conceptual model of study.

The conceptual model was used to test the following hypotheses (Figure 1).

H1 COVID-19 anxiety during pregnancy has effect on the maternal competency.

H2 COVID-19 anxiety during the postpartum has effect on the maternal competency.

H3 COVID-19 anxiety during pregnancy has effect on the maternal-infant attachment.

H4 COVID-19 anxiety during the postpartum has effect on the maternal-infant attachment.

H5 maternal-infant attachment has effect on the maternal competency.

## 2 Method

This study was prospective longitudinal study that approved by the Ethics Committee of Alborz University of Medical Sciences with code (IR.ABZUMS.REC.1399.235). Participants were selected from the health centers of Iran University of medical sciences that had the most clients and were the middle class. Participants were received written and oral information about the study, and written informed consent was obtained from them. They were free to decline participation or to withdraw at any time of study. All methods were performed in accordance with the relevant guidelines and regulations.

### 2.1 Study population

This study was conducted on 253 pregnant women in the third trimester of pregnancy who were referred to the health centers of Iran University of medical Sciences from January to July 2021 and met the study inclusion criteria include: Pregnant women during the COVID-19 pandemic and the third trimester of pregnancy (weeks 28–40), having no history of disabilities. The Mothers with a history of psychiatric disease. Mothers with high-risk pregnancies (placental abnormalities, diabetes, hypertension and other complication), mothers with infants with congenital anomalies and mothers with COVID-19 are excluded from study.

The participants were selected using the multi-stage sampling. In the first stage, the health centers of Iran University of medical sciences from each region as districts (clustered sampling) were selected then we consider health centers that had the most clients and were the middle class based on zoning of the municipality (simple random sampling). In the third stage, the eligible pregnant women through integrated health system were selected using the convenience sampling. Given the association between COVID-19 anxiety and maternal competency, the Pearson's correlation coefficient is at least (r = 0.2) [16]. With a 95% confidence interval and a 90% power, using the following formula, the required sample size was 253. The sample size was calculated 300 based on the 15% design effect

$$n={\left(\frac{z_{1-\alpha /2}+Z_{1-\beta }}{0.5\times ln\left(\frac{1+r}{1-r}\right)}\right)}^{2}+3$$

First, a list of pregnant women was prepared, and then the researcher contacted the eligible women. The study objectives were explained to them. The demographic questionnaire and the COVID-19 anxiety scale were completed in third trimester of pregnancy. The pregnant women were followed up until the delivery to assess the pregnancy and labor complications. After that, the COVID-19 anxiety scale, Müller's mother-infant attachment questionnaire were completed at 48-hour and maternal competency questionnaire were completed at 6-week after delivery. The questionnaires in the Google form format were completed by the social media platform, and also in case the mothers did not have access to social networks, they were asked to present to complete the questionnaires by the coordination by phone.

### 2.2 Questionnaires

#### 2.2.1 Corona disease anxiety scale (CDAS)

Corona disease anxiety is defined as a feeling of worry, anger, or dissatisfaction with Covid 19 with an uncertain outcome, this questionnaire was used during pregnancy and post -partum. Corona-related anxiety is an 18-item tool on a Likert scale from zero to 3. Each participant receives a score from 0 to 54. it measure corona-related anxiety in two dimensions, psychological and physical symptoms. The validity and reliability of this questionnaire have been assessed in Iran by Alipour et al. Moreover Cronbach's alpha coefficient for psychological symptoms as (α = 0. 879), physical symptoms as (α = 0. 861), and for the whole questionnaire as (α = 0. 919) [17]. In this study we assess qualitative and quantitative methods to determine the content validity of the survey again and confirmatory factor analysis (CFA) using Partial lease square 3(PLS3) for determining construct validity, results of CFA showed two question (1. I am concerned about transferring the virus to others and 2. I have lost my appetite because of thinking about Coronavirus) had factor loading below 0.7, and these two questions were deleted from questionnaire for entering the designed model. In this study we use the total score o CDAS in model.

#### 2.2.2 Müller Maternal Attachment index (MAI)

The MAI measures of maternal affectionate attachment. Müller Maternal Attachment index (MAI) was developed by Müller in 1994. It is contains 26 items that are scored from almost never [1] to almost always [4]. The range of scores is between 26−104, which high scores indicate a high degree of attachment of the mother to the baby [18]. Its validity has been approved through content validity and its reliability has been approved with a Cronbach's α of 0.89 In Iran [19].

#### 2.2.3 Parenting sense of competence scale (PSOC)

The **PSOC** in this study measures new mothers' beliefs, values, and perceived skills regarding being a mother. Gibaud-Wallston [20]designed PSOC, which contains 17 items with a five-point Likert scale (1 – completely disagree, 6 – completely agree), which the higher scores represent higher parental competence. The reliability and validity of the scale were determined by Abdollahpour et al in Iran [21].

### 2.3 Statistical analysis

Statistical analysis was performed using SPSS version 16. Also, the normality of the data was determined by the skewness and kurtosis, and the missing data were determined and replaced with the median values. Smart Partial Lease Square version 3 (PLS) was used to analyze the data in order to determine the validity and reliability of the model and the relationship between variables. Smart PLS was run as the outer (measurement) model and inner (structural) model. First, confirmatory factor analysis was performed, and then the validity and reliability of the model, the fit and quality of the model were determined. Finally, the relations between the independent and dependent variables were determined by the Structure equation model (SEM). In this study, Covid-19 anxiety during pregnancy and Covid-19 anxiety during postpartum period were independent variables, mother infant attachment was mediating variables and maternal competency was dependent variable.

## 3 Results

The mean age of the study participants was (26.8 ± 5.5). 92.9% of the participants were housewives.78.1% of the participants had a high school diploma and a university degree. 41.1% of the participants had history of COVID-19 infection. The results of the present study demonstrated that the mean maternal competency scores of the participants did not differ significantly in terms of age, education level, occupation, mode of delivery, baby's gender, and number of pregnancies, but significantly differed in terms of pregnancy intention, economic status and social support (Table 1).

**Table 1:** Comparison of mean and standard deviation of the maternal competency.

Variable		F (%)	Mean ± SD	P-value		F (%)	Mean ± SD	P-value
Age				0.51∗	Gravity			0.54∗
Total competency	<22	63 (25.3)	9.7 ± 69.4		1	124 (49)	68.14 ± 9.6	
	22–27	76 (30.3)	9.6 ± 68.1		2	77 (30.4)	65.7 ± 11.4	
	27–32	74 (29.4)	9.7 ± 66.6		3	33 (13)	71 ± 6.6	
	32–37	28 (11.1)	10.09 ± 66.6		>4	19 (7.5)	68.5 ± 8.2	
	Over 37 years of age	10 (3.9)	11.5 ± 66.4					
occupation				0.74∗∗	Type of delivery			0.54∗∗
Total competency	Housewife	235 (92.9)	9.6 ± 68.1		NVD	108 (42.7)	67.8 ± 9.8	
	Employee	18 (7.1)	10.1 ± 66.5		CS	145 (57.3)	67.9 ± 9.8	
Education level				0.83∗	Social support			0.04∗
Total competency	High school	56 (22.1)	9.5 ± 66.3	0.23∗	Husband	135 (53.4)	69.2 ± 9.08	
	Diploma	125 (49.4)	9.8 ± 68.2		Nobody	3 (1.2)	65.7 ± 9.7	
	University	72 (28.5)	9.9 ± 68.9		Family	115 (46.6)	70.1 ± 12.8	
Infertility History				0.64∗∗	**Gender**			0.83∗
Total competency	Yes	12 (4.7)	34.7 ± 7.4		Boy	143 (56.5)	67.8 ± 9.8	
	NO	241 (95.3)	37.1 ± 4.9		Girl	110 (43.5)	68.08 ± 9.7	
Pregnancy intention		0.045∗∗			**Covid-infection**			0.44∗∗
Total competency	Yes	206 (81.4)	67.5 ± 9.4		Yes	104 (41.1)	68.4 ± 9.2	
	No	46 (18.2)	69.5 ± 11.4		No	149 (58.9)	67.5 ± 10.2	
Economic status				0.026∗	**Variables**	Mean ± SD		
Total competency	weak	51 (20.3)	64.5 ± 9.5		Anxiety before pregnancy	31.7 ± 9.1		
	good	172 (68)	68.2 ± 9.7		Anxiety post-partum	30.5 ± 9.8		
	excellent	29 (11.5)	70.3 ± 9.7		Total attachment	100.2 ± 4.7		
					Total competency	67.8 ± 9.8		

The results of independent t-test showed that there was no significant difference in the mean maternal competency scores of the participants with respect to pregnancy complications (such as diabetes, hypertension, preterm birth, postpartum hemorrhage and hospitalization in the neonatal intensive care unit (NICU).

### 3.1 The measurement model

In the measurement model, ten tests were assessed (confirmatory factor analysis, Cronbach's alpha, composite reliability, the Spearman's rank-order, The average variance extracted (AVE), The Cross-loading test, Fornell - Larcker test, Hetro trait mono trait (HTMT), The crossed validated communality (CV- COM) and SRMR).

The first test was confirmatory factor analysis (CFA), and items that had factor loadings below 0.7 were removed from the model [22]. Cronbach's alpha and composite reliability and the Spearman's rank-order correlation were used to estimate the reliability of the model. In all cases, values greater than 0.7 were acceptable [23] (Table2).

**Table 2:** Criterion of reliability of the model.

Variables	Cronbach's Alpha	Composite Reliability	RHO_A
Covid-19 anxiety during pregnancy	0.91	0.91	0.89
Covid-19 anxiety during postpartum	0.94	0.94	0.94
Maternal-infant attachment	0.77	0.83	0.77
Maternal competency	0.84	0.87	0.84

The average variance extracted (AVE) test was used to determine the convergent validity of the model. The AVE value of each variable should be higher than 0.5. In the second test of the convergent validity test, the values of the composite reliability of each variable should be greater than those of the AVE [24]. The test results showed that the AVE values were higher than 0.5 and the value of the Composite reliability for all variables was higher than that of the AVE, thus the model had good convergent validity.

The Cross-loading test, Fornell - Larcker test and Hetro trait mono trait (HTMT) criterion were used to assess the divergent validity. In the table showing Cross-loading, each item got the highest factor loading for the variable [25]. Table 3 shows the Fornell and Larcker test of divergent validity. As shown in Table 3, the square root of AVE for all variables was greater than the correlation of that variable with other variables. The results of HTMT test also showed that the relationship between the two variables was less than 0.9 and the divergent validity of the model was also confirmed (Table 3).

**Table 3:** Fornell-Larcker criterion, CV COM and SRMR index of model.

	Covid-19 anxiety during pregnancy	Covid-19 anxiety during postpartum period	Maternal competency	maternal-infant attachment	CV-COM	SRMR
Covid-19 anxiety during pregnancy					0.031	0.07
Covid-19 anxiety during postpartum period	0.613				0.41	
Maternal competency	0.175	0.131			0.23	
Maternal-infant attachment	0.111	0.14	0.56		0.27	

The crossed validated communality (CV- COM) index was used to assess the quality of the outer (measurement) model in terms of predictive power with values of 0.02, at 0.15 and 0.35, representing low, intermediate, and high quality, respectively [26]. Values of CV- COM for all the variables were greater than 0.15 (Table 3), so the measurement model had an intermediate predictive quality. And SRMR was lower than 0.08 that showed good quality of model.

### 3.2 The structural model

**Figure 2:**
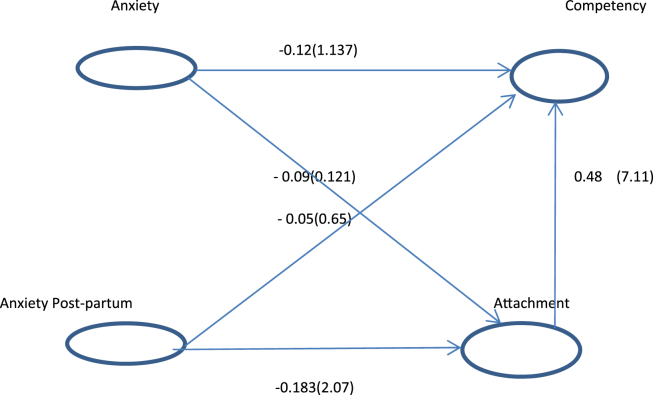
Path coefficient of variables.

The structural model was used to evaluate the relationship between the anxiety during pregnancy and postpartum period, the maternal-infant attachment and maternal competency. Figure 2 and Table 4 show the significant values and path coefficients of the study variables.

**Table 4:** Path coefficient and determined variance of structural equation model.

Hypotheses	β	T value	P value	Result
H1	−0.12	1.137	0.23	rejected
H2	−0.05	0.65	0.49	rejected
H3	−0.09	0.121	0.33	rejected
H4	−0.183	2.07	0.02	accepted
H5	0.48	7.11	0.000	accepted
**Explained variance of maternal competency**		R-squared		Adjusted R-squared
		0.25		0.24

The results of this study showed that the COVID-19 anxiety during pregnancy and postpartum period did not affect the maternal competency. Also, the COVID-19 anxiety during pregnancy had no effect on the maternal-fetal attachment. The results of the structural model showed the COVID-19 anxiety during postpartum period had a significant negative effect on the maternal- infant attachment (β = −0.183), If the COVID-19 anxiety during postpartum period changes by one unit maternal-infant attachment would change 0.183 in the reverse direction. Our results also showed that the maternal- infant attachment has a significant positive effect on the maternal competency (β = 0.48), If maternal-infant attachment changes by one unit, the maternal competency would change 0.48 in the same direction (Table4 and Figure 2). Based on R-square values, the maternal-infant attachment, and COVID-19 anxiety during pregnancy and postpartum period could predict 25% of the parental competence (Table 4).

This value is a moderate value based on the three values of Chin [27]. 0.19,0.33, and 0.67 Also, to determine the fit of the model, the Standardized Root Mean Square Residual (SRMR) index was used, which according to Hensler [28], it should be less than 0.08, and in this study, it was 0.07, which showed that the model has a good fit.

To assess the mediating role of maternal-infant attachment, the model was performed without the presence of a mediating variable (attachment) and with the presence of the mediating variable. Given the lack of statistical significance of model path coefficients in the absence of a mediator, COVID-19 anxiety during pregnancy and postpartum period showed no significant effect on the maternal competency, so mother-infant attachment was not considered as a mediator according to the Sobel test [29].

## 4 Discussion

The results of the present study demonstrated that pregnancy intention, economic status and social support could affect the maternal competency. Our results also showed that the mean maternal competency scores of the participants did not differ significantly in terms of age, education level, and occupation, mode of delivery, baby's gender, and number of pregnancies. A study showed that several factors, such as age, socioeconomic status, the attitudes towards childbearing, previous pregnancy, maternal and infant health status, family functioning, social support, and stress could contribute to the maternal acceptance [30]. In this study, the mean age of the participants was the appropriate age to become a mother, and most of them had a high school diploma, university degree, and most of them were housewives who were suitable for the motherhood. Given that this study was conducted during the COVID-19 pandemic, and the problems of home quarantine were the same for all mothers, it could reduce the effect of demographic characteristics.

The results of the present study showed that the mean score of maternal competency was higher in mothers who had social support from family (Table 1). In a study conducted in Thailand in 2020, perceived social support showed a significant positive correlation with the maternal competency, and the most important factor was also the transition to motherhood and the maternal competency [31]. Also, a study performed in 2021 in Denmark showed that the lack of social support and poor mental health of mothers had a negative effect on the maternal competency, leading to the poor mother-infant relationship [32]. According to study results, Social support facilitates the transition to motherhood, social support, including the positive support of family and friends, could increases parenting competence by providing encouragement and resources during the period of role-transition.

The results of the present study showed that the mean score of maternal competency varied based on pregnancy intention, and the mean score of maternal competency in mothers who wanted to become pregnant was higher than that in those who had an unwanted pregnancy. The results of a study demonstrated those mothers with low pregnancy competency had higher levels of anxiety, and the maternal attachment and maternal acceptance were lower in them, and also pregnancy acceptance could predict 15% of maternal competency [33] Also, our findings were consistent with the results of a systematic review suggesting that one of the causes of problems in the maternal competency and the poor mother-infant relationship is the lack of readiness to be a mother and women's reluctance for pregnancy [34]. According to our results, women's readiness to be a mother and their willingness to become pregnant could play an important role in the maternal competency.

The results of the present study revealed that the mean score of COVID-19 anxiety during pregnancy was 31.7 ± 9.5. A study reported that the mean score of COVID-19 anxiety during pregnancy was 10.5 ± 8.51, which might be due to the time of sampling and the peak of the disease at different times [35]. The results of the present study showed that the COVID-19 anxiety during pregnancy had no effect on maternal - infant attachment and the maternal competency. A study in Italy in 2021 investigated the effects of perceived anxiety during pregnancy, and the COVID-19 anxiety during pregnancy on the maternal - infant attachment based on a structural model, and the results showed that perceived anxiety during pregnancy could affect the maternal -infant attachment, but COVID-19 anxiety during pregnancy did not have direct effect on the maternal-infant attachment, but perceived anxiety during pregnancy had a direct effect on maternal-fetal attachment [36], which were consistent with our results. In the present study, the maternal-infant attachment was measured at six-week postpartum, which due to the transition of time and prolongation of the COVID-19 pandemic might reduce the effect of the COVID-19 anxiety during pregnancy. It might also be due to quarantine and isolation of the mother and more contact with the infant that the COVID-19 anxiety during pregnancy showed no effect on the maternal-infant attachment and the maternal competency. And also in this study we measured only COVID-19 anxiety and there is not any correlation between COVID-19 anxiety during pregnancy and COVID-19 anxiety in post-partum, it is suggested that in future studies pregnancy's anxiety and effect of COVID-19 anxiety on it will be measure.

The results of the present study showed that the COVID-19 anxiety during the postpartum period had a significant negative effect on the maternal-infant attachment. Also, the COVID-19 anxiety during the postpartum period could indirectly affect the maternal-infant attachment and the maternal competency. A study performed in Japan indicated that perceived anxiety in the postpartum period could influence the maternal-infant attachment and feeing anger and rejection of motherhood role one month after childbirth [37]. By learning the skills necessary to care for their infant, women during the postpartum period could improve the maternal-infant attachment, leading to acceptance of their motherhood role and improve maternal competency. The lack of relationship between mother and infant, and the perception of infant care and perceived stress could affect the maternal competency [38]. The results of a study showed that there was an inverse relationship between the postnatal perceived stress and mother-infant interaction and the maternal competency, and also postpartum training programs could affect mother-infant interaction [39]. Results of model showed that COVID-19 anxiety during pregnancy and post-partum did not directly effect on maternal competency and it effects inversely on maternal-infant attachment during postpartum. And also maternal infant attachment effect on maternal competency during post-partum. Based on this results its suggested that after delivery, maternal- infant attachment will be considered specially during COVID-19 pandemic because anxiety of covid 19 might be effect in mothers and infants distance. Maternal- infant attachment and COVID-19 anxiety predict 25 % of maternal competency during pandemic; therefore we suggested more studies for determining other variables that impact maternal competency during pandemic.

One of the strengths of this study is that it was a prospective study, but one of the limitations of the study was the lack of measurement of maternal competency score in mothers with COVID-19, and only the history of COVID-19 in this study was questioned. Therefore, it is recommended that a cross-sectional study should be conducted to evaluate the maternal competency and the maternal attachment in mothers with COVID-19.

## 5 Conclusion

Mothers experience higher levels of the COVID-19 anxiety during pregnancy and postpartum; therefore, it is recommended that particular attention should be given to the psychological support of pregnant women during the COVID-19 pandemic and quarantine. Also, the COVID-19 anxiety during the postpartum period had a negative effect on the maternal-infant attachment and maternal competency, which necessitates the need for the support of mother-infant relationship and providing the online training to promote the maternal infant attachment patterns and maternal competency during the COVID-19 pandemic.

## Declarations

### Author contribution statement

Zahra Mirzaki: Conceived and designed the experiments; Performed the experiments; Wrote the paper.

Zahra Behboodi Moghdam: Analyzed and interpreted the data; Wrote the paper.

Mitra Rahimzadeh: Analyzed and interpreted the data;Fahimeh Ranjbar: Analyzed and interpreted the data; Wrote the paper.

Sara Esmaelzadeh-Saeieh: Conceived and designed the experiments; Wrote the paper.

### Funding statement

The study was approved by the Ethics Committee of Alborz University of Medical Sciences with code [IR.ABZUMS.REC.1399.235].

### Data availability statement

Data will be made available on request.

### Declaration of interest's statement

The authors declare no conflict of interest.

### Additional information

No additional information is available for this paper.
